# Ivabradine: a preliminary observation for a new terapeutic role in patients with multiple organ dysfunction syndrome

**DOI:** 10.1007/s00392-014-0722-2

**Published:** 2014-05-08

**Authors:** Vincenzo De Santis, Giacomo Frati, Ernesto Greco, Luigi Tritapepe

**Affiliations:** 1 Department of Clinical Physiology, UCL, London, UK; 2 Department of Medical-Surgical Sciences and Biotechnologies, Sapienza University of Rome, 04100 Latina, Italy; 3 Department of AngioCardioNeurology, IRCCS NeuroMed, Pozzilli, Italy; 4 Department of Cardiovascular, Respiratory, Nephrological, Anesthesiological, and Geriatric Sciences, Policlinico Umberto I-Sapienza University of Rome, 00161 Rome, Italy

Sirs:

Severe sepsis is a major cause of mortality and morbidity worldwide. Septic patients often develop multiple organ dysfunction syndrome (MODS) that is characterized by an acute functional impairment of two or more organs so that homeostasis cannot be maintained without intervention [1]. Patients with MODS typically present with an elevated heart rate (HR) that accompanies a highly impaired autonomic dysfunction with depressed parasympathetic control of the heart. An elevated heart rate was found to increase the incidence of major cardiac events in critically ill patients [2–4]. Furthermore, an elevated heart rate in the early phase of MODS was found to be an independent predictor of increased 28-day mortality [5].

As the currently available specific regimes for the treatment of MODS show only very limited therapeutic benefit compared with its high incidence and mortality, there is an urgent need for therapeutic innovations, targeted to improve the prognosis of MODS patients is mandatory.

Ivabradine is a pure heart rate-lowering drug that acts specifically on the sinoatrial node by selectively inhibiting the hyperpolarization-activated cyclic nucleotide (HCN) channel of cardiac pacemaker cells by entering and binding to a site in the channel pore from the intracellular side without affecting the other cardiac ionic currents.

Two very important prospective randomized trials have showed benefits derived from ivabradine administration in patients with stable coronary artery disease and in chronic heart failure patients [6, 7]. We previously reported a significant heart rate reduction in patients with catecholamine-induced tachycardia after high-risk cardiac surgery treated with ivabradine [8]. A prospective randomized trial testing ivabradine in MODS is currently under way [9].

The aim of this case study was to report the preliminary results of ivabradine use in three patients who developed sepsis related MODS after cardiac surgery.

The baseline characteristics are summarized in Table 1.

**Table 1:** Baseline characteristics

Variable	Patient no. 1	Patient no. 2	Patient no. 3
Age (years)	65	62	70
Sex	Male	Male	Male
Ejection fraction (%)	45	50	45
Acute renal failure	Yes	Yes	Yes
Apache II score	32	30	32
ACE inhibitor	Yes	Yes	Yes
β-blockers	Yes	Yes	Yes
Statins	Yes	Yes	Yes
Aortic cross clamp time (min)	100	97	110
CPB time (min)	128	112	135
Type of infection	Gram-positive	Gram-positive	Gram-negative
Site of infection	Endocarditis	Endocarditis	Lung

*CPB* cardiopulmonary bypass

A standardized anesthesia and postoperative sedation protocol was used for all patients. Routine monitoring included: standard 8-lead electrocardiogram, radial artery pressure, thermodilution pulmonary artery catheter, and transesophageal echocardiography evaluation.

Hemodynamic and metabolic outcomes were analyzed with a hierarchical linear model for repeated measurements to assess trends over time. Post-hoc comparisons between the estimated means at baseline and at the end of follow-up were investigated through suitable contrasts, for each outcome separately. A spatial power covariance structure was used to account for unequally spaced time occasions during the follow-up. All analyses were performed using SAS Statistical Package, Release 9.2 (SAS Institute, Cary, NC, USA). All the patients developed sinus tachycardia (HR >90 bpm). Mean arterial pressure (MAP) was sustained with norepinephrine support. MODS manifested at the third postoperative day in patient 1 and 2 while in patient 3 during the fourth postoperative day. Ivabradine was administered twice daily via a nasogastric tube (starting dose 10 mg followed by a maintenance dose of 5 mg/12 h) for 18 h.

**Fig. 1:**
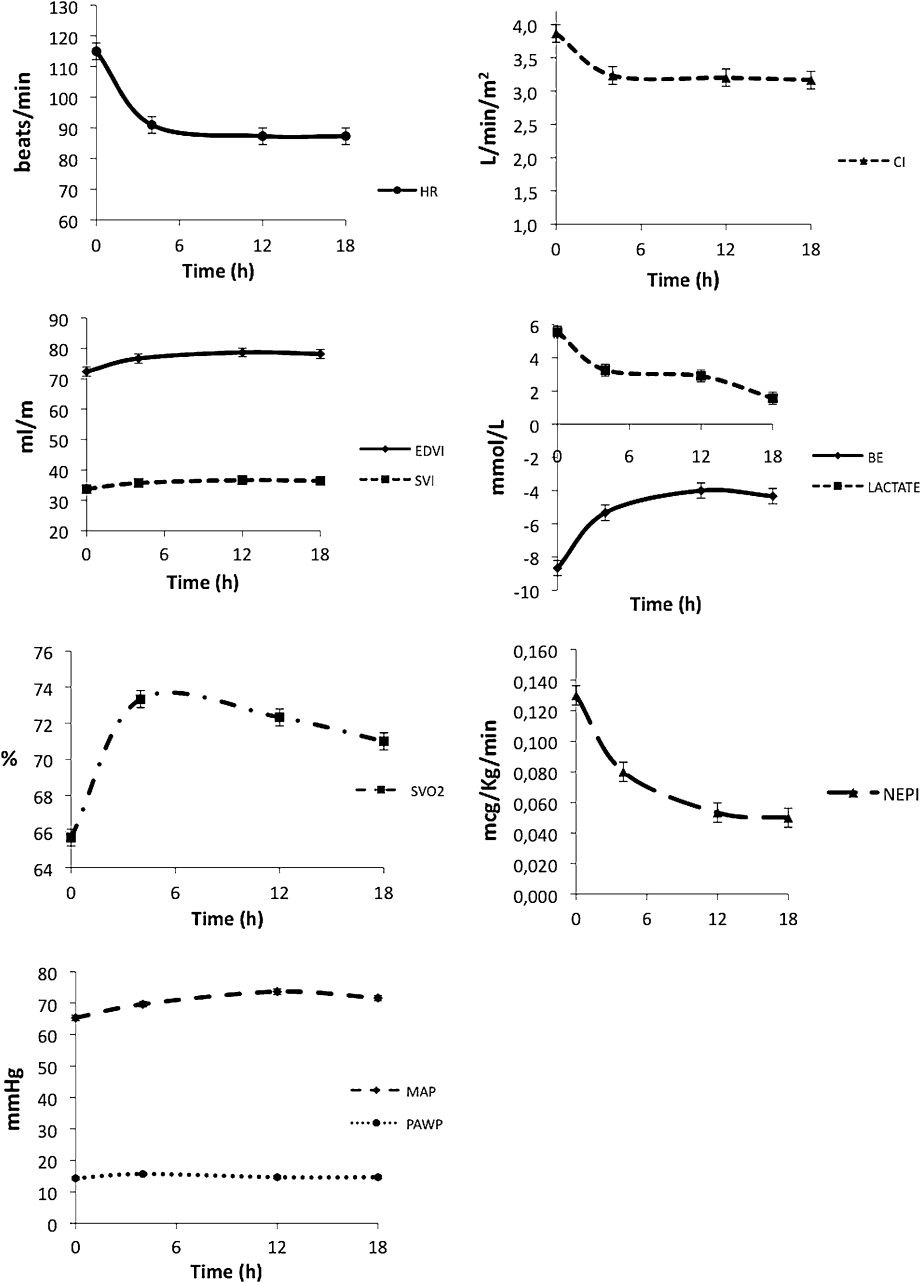
Longitudinal assessment of heart rate (HR), cardiac index (CI), end-diastolic volume index (EDVI), stroke volume index (SVI), base excess (BE), serum lactate (lactate), mixed venous saturation (SvO_2_), norepinephrine dose (NEPI), mean arterial pressure (MAP) and pulmonary capillary wedge pressure (PAWP). Data shown as mean and standard deviation

Hemodynamics, metabolic data and norepinephrine dosage are summarized in Fig. 1.

Heart rate (mean difference −27.6) and cardiac index (CI) decreased after ivabradine administration, whereas end-diastolic volume index (EDVI), stroke volume index (SVI), MAP and mixed venous saturation (SvO_2_) increased. Pulmonary capillary wedge pressure (PCWP),and right atrial pressure did not change over time. Serum lactate levels decreased, norepinephrine dose was reduced whereas base excess diminished.

Apache II score decreased after 24 h (31 vs. 27). All the patients survived and were discharged from the intensive care unit, respectively, on day 10 (patient no. 1), on day 12 (patient no. 2) and on day 18 (patient no. 3).

An elevated HR is a well established, independent and modifiable risk factor for cardiovascular events and mortality in critically ill patients [2]. Specifically autonomic dysfunction with an inadequately high HR occurs very frequently in MODS patients and is associated with a worse prognosis [4]. Sepsis-related tachycardia has several adverse effects on the heart, including restricted limited diastolic ventricular filling, increased oxygen requirements and potentially, a tachycardia-induced cardiomyopathy. Indeed, heart rate on presentation predicted survival in septic shock patients [10].

Ivabradine is a selective antagonist of the HCN channel. In vitro studies showed that endotoxin sensitized HCN channel for sympathetic stimulation, thereby increasing the heart rate [11]. Therefore, the negative chronotropic effect of ivabradine might be an interesting option in MODS patients with sinus tachycardia.

In our preliminary case study ivabradine was able to reduce HR and the three studied patients showed a concomitant increase in SVI, EDVI and SvO_2_. The hemodynamic improvement resulted in a consistent serum lactate level reduction and norepinephrine dosage.

Similar results are reported in a recent randomized trial of intravenous ivabradine in the context of acute ST-segment elevation myocardial infarction. Ivabradine produced a rapid and reversible slowing of HR not associated with changes in blood pressure or major side effects [12].

Although there are many drugs with negative chronotropic effect, such as calcium-channel blockers and β-blockers [13], they also possess strong negative inotropic effect and thus might be potentially harmful in most MODS patients. Ivabradine reduces heart rate by prolonging diastole, while the negative ino-tropic action of β-blockers prolongs both systole and diastole. As a result, for the same reduction in heart rate, ivabradine produces a greater prolongation of diastolic time than β-blocker [14]. Accordingly in our study, although not a comparative trial, an improvement of the diastolic function has been attested by the increase of the EDVI.

Septic shock has many of the manifestations of a hyperadrenergic response such as sympathetic neural and humoral activation, catecholamine and cortisol release into the bloodstream. The clinical and physiologic manifestations of these responses include tachycardia. Based on this logic, in a recent single-center randomized clinical trial Morelli et al. [15] reported that the open-label use of the short-acting β-blocker esmolol was able to achieve reductions in heart rate to target levels, without an increase in adverse outcomes compared with standard treatment.

Limitations of our study include the lack of power due to the small sample size and also the lack of a randomized group. In light of this weakness we preferred not to give particular emphasis to our findings in terms of *p* values, although the statistical methods used borrow strength from the repeated measures design. In our view these findings should be confirmed on a larger sample size.

Pharmacokinetic data available after oral administration of ivabradine in healthy persons demonstrated that after a 5, 10 or 20 mg single dose, a significant reduction in HR at 2 h postdose was observed in the highest dose group, whereas after repeated doses, a significant reduction in HR was observed from 2 to 4 h postdose for all three groups [16].

Another limitation of our study is that we were unable to measure the ivabradine plasma concentration and therefore, demonstrate drug absorption. However, since the incidence of gastrointestinal dysfunction after cardiac surgery is very low we can reasonably exclude that the treated patients might have not absorbed the drug [17].

In conclusion our study, albeit preliminarily, demonstrated that ivabradine administration was well tolerated and, produced HR reduction in MODS patients. The results of the MODI(f)Y trial are needed to confirm our findings.
